# The hnRNP-like Nab3 termination factor can employ heterologous prion-like domains in place of its own essential low complexity domain

**DOI:** 10.1371/journal.pone.0186187

**Published:** 2017-10-12

**Authors:** Travis J. Loya, Thomas W. O’Rourke, Daniel Reines

**Affiliations:** Department of Biochemistry, Emory University School of Medicine, Atlanta, GA, United States of America; John Curtin School of Medical Research, AUSTRALIA

## Abstract

Many RNA-binding proteins possess domains with a biased amino acid content. A common property of these low complexity domains (LCDs) is that they assemble into an ordered amyloid form, juxtaposing RNA recognition motifs in a subcellular compartment in which RNA metabolism is focused. Yeast Nab3 is one such protein that contains RNA-binding domains and a low complexity, glutamine/proline-rich, prion-like domain that can self-assemble. Nab3 also contains a region of structural homology to human hnRNP-C that resembles a leucine zipper which can oligomerize. Here we show that the LCD and the human hnRNP-C homology domains of Nab3 were experimentally separable, as cells were viable with either segment, but not when both were missing. In exploiting the lethality of deleting these regions of Nab3, we were able to test if heterologous prion-like domains known to assemble into amyloid, could substitute for the native sequence. Those from the hnRNP-like protein Hrp1, the canonical prion Sup35, or the epsin-related protein Ent2, could rescue viability and enable the new Nab3 chimeric protein to support transcription termination. Other low complexity domains from RNA-binding, termination-related proteins or a yeast prion, could not. As well, an unbiased genetic selection revealed a new protein sequence that could rescue the loss of Nab3’s essential domain via multimerization. This new sequence and Sup35’s prion domain could also rescue the lethal loss of Hrp1’s prion-like domain when substituted for it. This suggests there are different cross-functional classes of amyloid-forming LCDs and that appending merely any assembly-competent LCD to Nab3 does not restore function or rescue viability. The analysis has revealed the functional complexity of LCDs and provides a means by which the differing classes of LCD can be dissected and understood.

## Introduction

In *Saccharomyces cerevisiae*, a specialized termination machinery ensures the proper termination of short, non-coding transcripts by RNA polymerase II (Pol II) [1]. This complex depends upon the Nrd1-Nab3-Sen1 (NNS) complex [2]. Nrd1 and Nab3 are hnRNP-like proteins that dimerize to bind specific RNA sequence elements *via* their RNA recognition motifs (RRMs) and associate with Pol II through Nrd1’s C-terminal interaction domain (CID) [3, 4]. Sen1, a putative helicase, is thought to join this ribonucleoprotein complex during Pol II elongation, thereby terminating transcription [5]. Prior work in our lab established that a C-terminal segment of Nab3 is essential for viability, and is important for proper termination in both a reporter assay as well as at an endogenous target of NNS, the *IMD2* gene [6]. This Nab3 domain is made up of a low complexity amino acid sequence that is rich in glutamine (Q) and proline (P), intrinsically disordered, and amyloid-like based on computational models and biochemical data. A combination of electron microscopy, thioflavin T-binding, circular dichroism, and semi-denaturing detergent agarose electrophoresis (SDD-AGE) has shown that Nab3’s low complexity domain, as well as full length Nab3, form authentic amyloid fibrils [7]. Furthermore, glutamine to glutamate mutations in the Nab3 LCD abrogate amyloid formation and the mutant protein no longer supports cell viability [8]. Nab3 also has an amino-terminal, low complexity region with long tracts of aspartate (D) and glutamate (E) residues whose function is unknown. The segment is dispensable for cell viability [3] and it is not expected to self-assemble into an amyloid state based on its high charge density. As well, this D/E domain does not score as prion-like, as observed for the C-terminal Q/P domain which will be the focus of this report.

A previously characterized genetic interaction has shown that different mutants of *NAB3* can complement each other in *trans* to restore viability, suggesting multiple copies of Nab3 are present at termination complexes. Nab3 was also shown to interact with itself in living yeast in a protein fragment complementation assay [7, 9]. As well, multiple Nrd1 and Nab3 recognition sites are found in transcripts terminated by the NNS system, again suggesting multiple copies of the proteins work together *in vivo* [10]. These findings suggest that the LCD contributes to the functionally important assembly of the Nab3 termination factor with itself or other partners. Through its role in RNA biogenesis, the NNS complex is a key regulator of transcriptome changes seen during nutrient stress, showing increased recruitment of Nab3 to genes involved in energy usage and cell growth as well as a decrease in binding to genes regulating the stress response [11–13]. Concomitant with this shift is the movement of Nab3 and Nrd1 to a perinuclear subcellular structure [11]. How this particle assembles, dissociates, and functions in RNA metabolism is poorly understood.

There is a noted overrepresentation of LCDs in RNA-binding proteins which is thought to reflect their functional importance [14, 15]. Low complexity domains contain stretches of amino acids with limited sequence diversity that are often intrinsically disordered. However, under some conditions, LCDs can attain very stable ordered secondary structures, often β-sheets, which assemble into intracellular amyloid aggregates. LCDs in prions are important for their ability to aggregate and become heritable particles. The biology of prion proteins is under active investigation and domains of low complexity which often appear prion-like in their composition have received considerable interest due to their implication in numerous disease states [16, 17], their frequent occurrence in RNA-binding proteins [14, 15], and their participation in the formation of non-membrane bound organelles in both the nucleus and cytoplasm [15, 18–20]. The hallmark of many neurodegenerative disorders is the formation of insoluble protein aggregates in neurons, many of which contain proteins with low complexity domains [21].

Here we have analyzed the functional role of the prion-like LCD of yeast Nab3, which can assemble into a robust amyloid form, and is essential for cell viability. We show that the Nab3 LCD can be exchanged for the low complexity regions of other proteins including those from a bona fide prion, Sup35, or another RNA-binding protein, Hrp1. Although they substitute functionally, these domains lack strong primary sequence homology to the natural Nab3 LCD they replace. Furthermore, amino acid content does not necessarily correlate with their effectiveness. Using the essential nature of the LCD as a genetic tool, we also selected from a pool of bovine DNA, sequences that can functionally replace the natural domain. Taken together, these findings indicate a remarkable degree of versatility between LCDs, however, not all LCDs are capable of complementing function, showing that there is specificity within the larger family of low complexity regions. We also show that Nab3’s C-terminus is more complex than previously appreciated, as it can be subdivided into two regions, a Q/P-rich segment and a small piece with structural homology to an α-helix found in human hnRNP-C. The essential nature of Nab3 and its C-terminal region have provided a genetic means of probing the role of low complexity domains in RNA-binding proteins.

## Materials and methods

### Plasmid construction

Nab3 chimeras were generated by cutting pRS315-Nab3 [22] with *Nde*I and *Xho*I and inserting similarly cut PCR products encoding heterologous LCD’s amplified with the primers listed in Supporting Information, S1 Table. The Nab3-Ent2 chimera was made by inserting synthetic DNA (GenScript, Piscataway, NJ) encoding the 60 amino acid core prion-like domain [23] into the *Nde*I and *Xho*I sites of pRS315-Nab3. The pRS315-Nab3hrp1scr plasmid was made by inserting synthetic DNA (GenScript) encoding a randomization of the 60 amino acid core prion-like domain of Hrp1 (YGNGMMGQTGNYGDGGMNFNNDNGQQNYMNGPEQQGYMGMNYMRPMQNQFYANQKMNPRNQY) into *Nde*I and *Xho*I-cut pRS315-Nab3. pRS315Nab3Δ134α was made by deleting DNA encoding Nab3 amino acids 669–784 in pRS315Nab3 using 5’-aggtggttgaggaggcggacc-3’ and 5’-cctgctggcaataatgttcaaagtctatta-3’. pRS315Nab3Δ134αL800A was made from pRS315Nab3Δ134α by Phusion mutagenesis using 5’-gatagtttagcaaaagcacaaaaatagactc-3’ and 5’-taatagactttgaacattattgccagc-3’. pRS315-Hrp1 was made by inserting a PCR product encoding *HRP1* (5’-ggatcctaggattgaacagtttcgac-3’ and 5’-ctcgagtacagctacttcaagtagag-3’) into pRS315 after both were cut with *Bam*HI and *Xho*I. pRS315-HRP1coredel was made by Phusion deletion mutagenesis of pRS315-HRP1 using 5’-aagcaaactggtatggattatactc-3’ and 5’-tgatgatttttgttgcatatgtc-3’. pRS315-Hrp1Sup35, pRS315-Hrp1coredelCT25, and pRS315-Hrp1-linker were made by inserting synthetic DNA (GenScript) encoding either the Sup35 N-domain residues 2–114, the CT25-encoded peptide (VCTCLCMSLCLKKGVKDTRQVVDDHLEG), or a peptide derived from pET32a (GSGSGHMHHHHHHSSGLVPRGSGMKETAAAKFERQHMDSPDLGTDDDDKAMADIGSEFEL), respectively, in place of the 60 amino acid core prion-like domain of Hrp1 (residues 329–388).

A library of plasmids with bovine genomic DNA fused to the Nab3 sequence was made by digesting pRS315-Nab3 with *Nde*I and *Xho*I, which releases a fragment encoding the C-terminal 191 amino acids of Nab3. Calf thymus DNA (Worthington) digested with *Nde*I and *Xho*I was gel-purified, ligated to the plasmid DNA and transformed into *E*. *coli* that was plated on solid medium. Colonies were pooled and plasmid DNA was prepared and used to transform yeast strain DY3111 (which contains a deletion of chromosomal *NAB3* and a plasmid bearing wildtype *NAB3* on a *URA3*-marked plasmid) to leu^+^. Transformants were selected on SC ura^-^ leu^-^ and moved to SC leu^-^ to allow for loss of the *URA3*-marked plasmid with wildtype *NAB3*. Cells were plated on fluoroorotic acid (FOA)-containing medium and colonies were re-streaked to FOA for purification. The resulting plasmid was rescued from yeast and sequenced. A number of parental plasmids containing full length *NAB3* were identified and excluded. Bovine DNA from clone CT25 was mapped as an exact match to chromosome 19 from 25435293–25436099 on the Btau4.6.1 assembly at www.bovinegenome.org using BLAST [24].

pRS306-nab3Δ134 was made by inserting the nab3Δ134 sequence released from pRS315-nab3Δ134 [22] by digestion with *Bam*HI and *Xho*I into similarly cut pRS306 [25].

### Yeast strains

Yeast strains used in this study are presented in Supporting Information, S2 Table. Cells were grown in rich medium (YPD), synthetic medium (SC), or standard selective drop-out medium (SC ura^-^, SC leu^-^ or SC ura^-^ leu^-^; [26]) at 30°C unless otherwise indicated. Plasmids were transformed into yeast strains using the lithium acetate method [27]. DY3111 was transformed with pRS315-*NAB3*, pRS315-*nab3*Δ*191STOP*, pRS315-Nab3Rat1, pRS315-Nab3Pcf11, pRS315-Nab3Rnq1, pRS315-Nab3Sup35, pRS315-Nab3Hrp1, pRS315-Nab3Hrp1scr, pRS315-Hrp1-linker, pRS315-Nab3-CT25, pRS315-Nab3ENT2LCD, pRS315-Nab3Δ134, pRS315-Nab3Δ134α, or pRS315-Nab3Δ134αL800A to generate strains DY351, DY3183, DY3184, DY3185, DY3186, DY4002, DY3193, DY3213, DY4500, DY4001, DY3244, DY3134, DY353, and DY377, respectively. The resulting strains were grown at 30°C on SC leu^-^ ura^-^. For plasmid shuffling, these strains were grown on SC leu^-^ followed by selection on FOA. This generated the following strains with only a single, *LEU2*-marked, plasmid: DY3182, DY3196, DY3245, DY4004, DY4006, DY359, and DY379. GFP reporter plasmids were transformed into DY3182, DY3196, DY3245, DY4004, DY359, DY379, to yield strains DY3187, DY3197, DY3246, DY4014, DY3912, and DY3913, respectively. DY3217 and DY3218 were made by transforming DY3034 with pREFGFPKan and pGALGFPKan respectively.

For tetrad analysis, the diploid YH990 [28] served as a starting strain for the ‘pop-in/pop-out’ [26] introduction of *nab3*Δ*134* into one of the two *NAB3* loci, generating strain DY3210. Conversion was confirmed by PCR for the two *NAB3* alleles. The diploids were sporulated and dissected.

The strain PSY818 (a gift from Dr. P. Silver, [29]), disrupted for chromosomal *HRP1* contains the essential *HRP1* gene on a *LEU2*-based plasmid. The *LEU2*-based plasmid was transformed with a *URA3*-based plasmid by transformation with pRS316-HRP1 to yield DY307 and screened for the loss of the *LEU2*-marked plasmid, to yield strain DY308. This strain was used as a parent to transform the plasmids pRS315-Hrp1coredel, pRS315-Hrp1coredel-CT25, pRS315-HRP1, pRS315-Hrp1coredel, pRS315, or pRS315-Hrp1Sup35LCD to yield strains DY318, DY387, DY388, DY389, DY1638, and DY3242, respectively. Loss of the *URA3*-marked copy of *HRP1* from these strains *via* shuffling yielded the cognate viable strains DY387F and DY3243.

### Flow cytometry

Cells were grown in SC ura^-^ leu^-^ in which glucose was replaced by galactose as the carbon source and grown overnight. Overnight cultures were diluted to an OD_600_ of 0.2 and allowed to grow to OD_600_ of approximately 1.0. Flow cytometry was performed using an LSRII (Becton-Dickson) with an excitation wavelength of 488nm and data was collected using FACSDiva software (Becton-Dickson). Analysis of results was performed using FlowJo ver. 10.1.

### Western blotting

Cells were collected, washed with water, and boiled for 5 min in loading buffer [30] before being resolved on a 6% polyacrylamide gel. Proteins were electrophoretically transferred to nitrocellulose which was blocked with 5% nonfat dry milk [31], and probed with antibodies against Hrp1 (gift from Dr. Michael Henry, [29]) or Nab3 (2F12-2; Dr. M. Swanson [4]). Signal was detected using horse radish peroxidase-conjugated anti-rabbit and anti-mouse IgG (Sigma Chemical Co.), respectively, using enhanced chemiluminescence performed in 100mM Tris pH 8.5, 1.25 mM luminol, 200nM p-coumaric acid, and 0.01% H_2_O_2_. Chemiluminescence was captured by exposure to X-ray film.

### Semi-denaturing detergent agarose electrophoresis

Purified protein samples were adjusted to 2% SDS, 140 mM β-mercaptoethanol, 10% glycerol, 0.002% bromophenol blue, 80 mM Tris, pH 6.8, incubated at room temperature for 10 min. (unless otherwise indicated), and separated by electrophoresis in agarose gels (1.5% w/v) in 40 mM Tris-acetate, pH 7.8; 1 mM EDTA, 0.1% SDS run at 4°C. Bio-Rad Precision Plus Protein Kaleidoscope (Cat. No. 161–0375) molecular weight markers were run as standards. Proteins were blotted to Protran nitrocellulose transfer membrane (Whatman) by capillary action for 18 hrs. Filters were blocked in 5% (w/v) nonfat dry milk in Tris-buffered saline with 0.1% Tween-20, probed with anti-Hrp1 antibody, and detected with chemiluminescence as described above.

## Results

### Some chimeric Nab3 proteins with heterologous LCDs support viability

**Fig 1:**
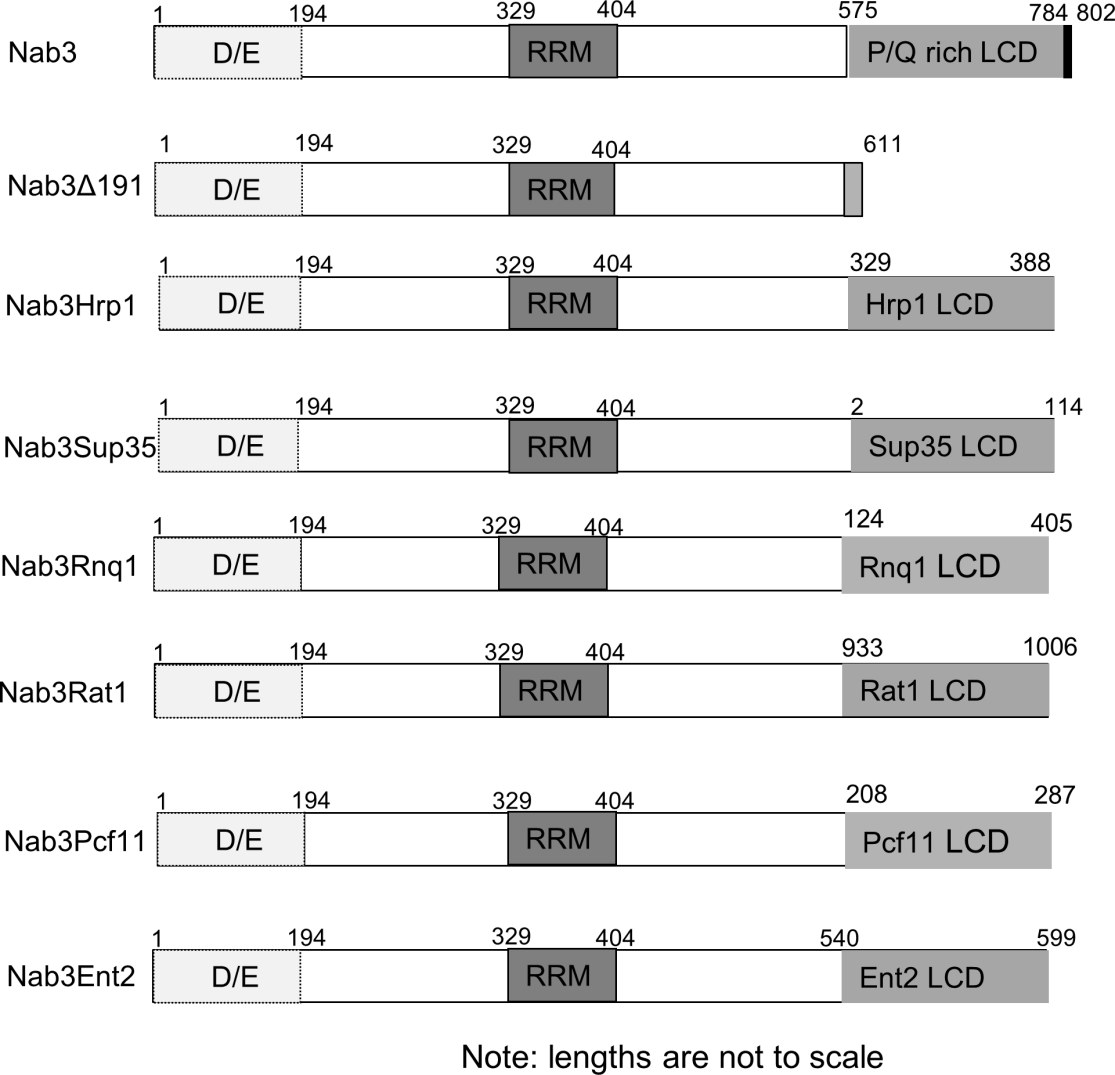
Schematic of Nab3 chimeras. The D/E-rich, RRM, and Q/P-rich domains of Nab3 are indicated. The positions of the amino acids derived from the respective heterologous proteins are indicated. Nab3’s human hnRNP-C structural homology domain (784–802) is indicated in black at the C-terminus.

**Fig 2:**
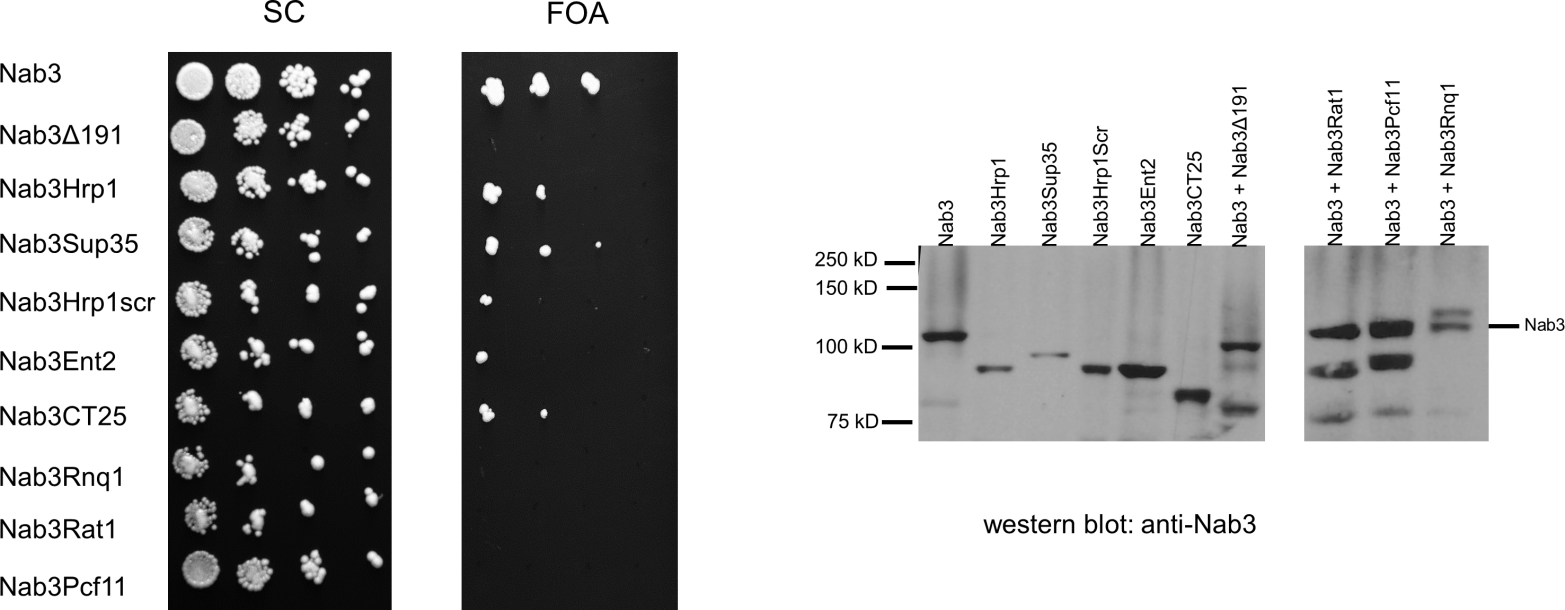
Growth and protein expression in yeast strains with Nab3-chimeras. Yeast cultures were diluted to 0.05 OD_600_ and serially ten-fold therefrom. Ten μl were spotted onto a single plate of the indicated solid media, and incubated at 30°C. From the top down, the strains expressed wildtype Nab3 and a second Nab3 protein, either wildtype Nab3 (DY351), the Nab3Δ191 ‘stem’ (DY3183), the Nab3Hrp1 chimera (DY3193), the Nab3Sup35 chimera (DY4002), the Nab3Hrp1scrambled chimera (DY3213), the Nab3Ent2 chimera (DY3244), the Nab3CT25 chimera (DY4001), the Nab3Rnq1 chimera (DY3186), the Nab3Rat1 chimera (DY3184), or the Nab3Pcf11 chimera (DY3185). The right side of the figure is a western blot of whole cell lysates from logarithmically growing strains containing the indicated Nab3 protein (left to right: DY3033, DY3196, DY3182, DY4006, DY3245, DY4004, DY3183) or wildtype Nab3 protein with an additional Nab3 derivative that could not support growth on its own (DY3184, DY3185, DY3186) lysed in sample buffer and subjected to SDS-PAGE and western blotting with an antibody against Nab3, as described in Methods. Each of the two western blot panels were run on separate gels and processed separately.

The Nab3 RNA-binding protein contains an essential, carboxy-terminal segment that includes a low complexity domain that is Q/P-rich ([3, 4, 22] Fig 1 and S1 Fig). The domain scores as prion-like using a prediction algorithm trained with authentic yeast prions [23]. Nab3 functions as a multimer in binding nascent RNA, leading to the hypothesis that the essential function of the LCD is to enable the assembly of Nab3, and possibly other proteins, as they work to terminate transcription across the genome [3, 22]. To further test this idea, and learn if simply any interaction domain could substitute for Nab3’s LCD, we prepared a lethal truncated version of Nab3 (Fig 1, Nab3Δ191) that is deleted for most of its LCD. This derivative, expressed from the *NAB3* promoter, was used as a stem onto which we grafted other prion-like domains in place of the natural sequence (Fig 1). Using as a starting point the Alberti *et al* [23] catalog of yeast proteins with prion-like domains, we tested: 1) prion-like LCDs from three termination-related yeast RNA-binding proteins Hrp1, Pcf11, and Rat1, 2) LCDs from two well-established cytoplasmic prion proteins, Rnq1 and Sup35, that are unrelated to transcription, and 3) an LCD from a membrane-interacting protein (Ent2) that has a high score as prion-like [23]. These chimeric Nab3 proteins were encoded on *LEU2*-marked plasmids which were individually introduced into a yeast strain in which chromosomal *NAB3* was disrupted, and which contained a covering *URA3-*marked plasmid with wildtype *NAB3*. The ability of different isolates to survive by virtue of their use of the resulting chimeric Nab3 proteins, following the loss of the wildtype *NAB3*-*URA3* plasmid, was tested on media containing FOA. Surprisingly, some, but not all, of these alternative prion-like sequences, supported cell viability when grafted onto Nab3, although growth rates were slower than wildtype (Fig 2). These included the prion-like region from the transcription termination factor Hrp1, the prion domain from the well-studied Sup35 protein, and the core prion-like domain from the membrane-associated cytoplasmic protein Ent2 (Fig 2 and S1 Fig). Fusions to Rat1, Pcf11, or Rnq1’s prion-like domains, all of which have been previously observed to autonomously form amyloid filaments, and one of which, Rnq1, is a *bona fide* prion, did not substitute for Nab3’s endogenous LCD in this plasmid shuffle regimen. Western blots using an anti-Nab3 antibody, confirmed that the resulting strains bearing the LCD-fusion partners from Sup35 (Nab3Sup35), Hrp1 (Nab3Hrp1), or Ent2 (Nab3Ent2) contained only the chimeric proteins as their sole source of Nab3 (Fig 2). The chimeric proteins that did not support viability accumulated to levels similar to wildtype Nab3 in the strains that bore both the wildtype and chimeric plasmid (Rat1, Pcf11, Rnq1; Fig 2), eliminating the possibility that these Nab3-derivatives failed to support growth because they were malformed and degraded. It is also noteworthy that, while the 60 amino acid Hrp1 prion-like domain can substitute, the large (282 amino acid) extended prion domain of Rnq1 could not reconstitute Nab3 function. Thus, the length of the added LCD is not positively correlated with function in this assay.

Work on yeast prion proteins has shown that the primary amino acid sequence of the glutamine- or asparagine-rich prion domains is less important for prion formation than their overall composition [32, 33]. This is seen in experiments in which the primary sequence can be scrambled and yet it remains capable of supporting prion formation [32, 33]. To test if the Hrp1 LCD, which substituted for Nab3’s LCD, could be scrambled and still retain function, we subjected the order of the amino acids to randomization which generated a version with an overall amino acid composition identical to the wildtype sequence (S1 Fig), and appended it to the Nab3 stem. As seen previously for other yeast prion-domains, Hrp1’s LCD could be scrambled and support cell survival (Fig 2).

**Fig 3:**
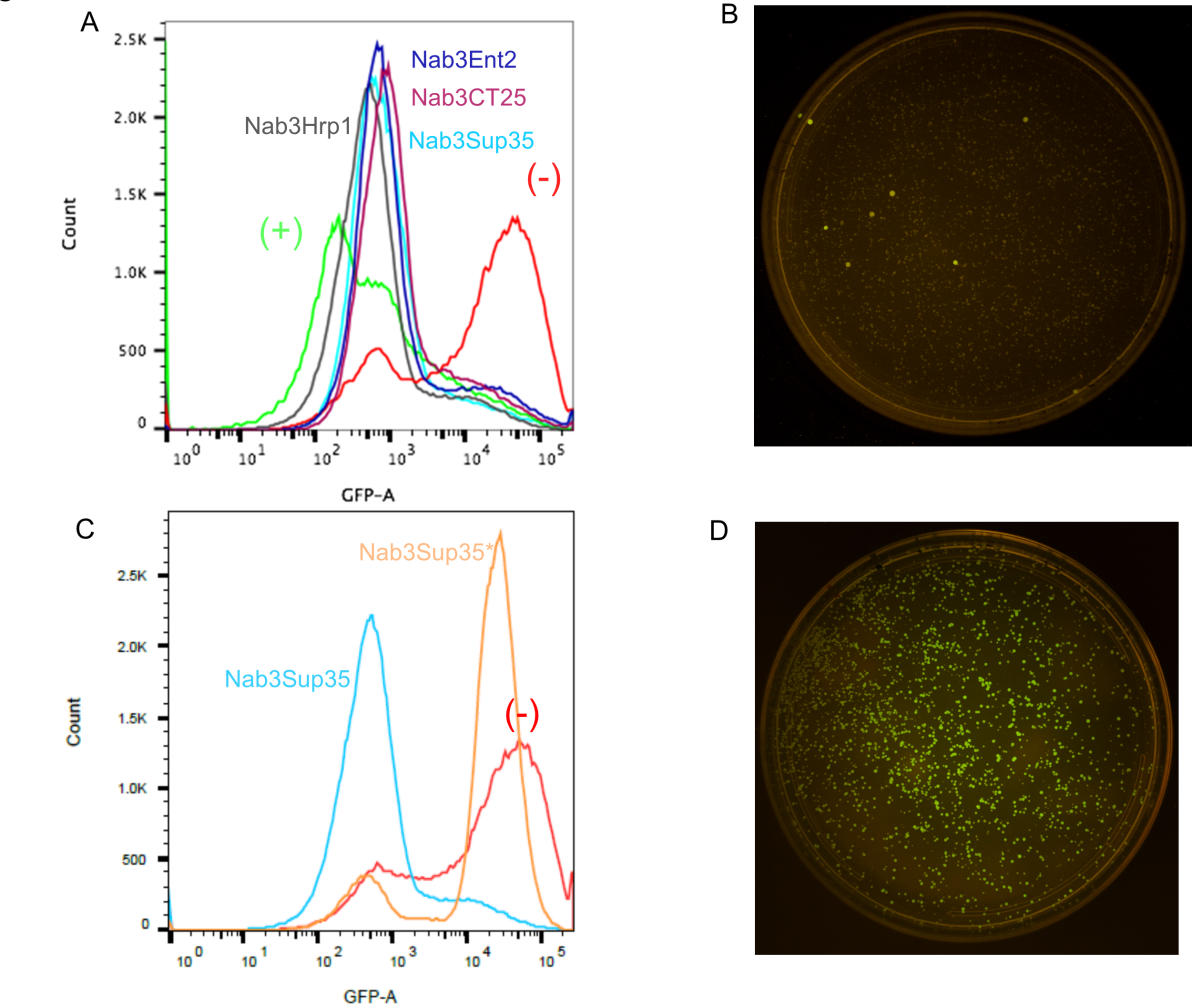
GFP expression in strains with chimeric Nab3 proteins. (A) Flow cytometry profiles for control and experimental yeast strains. A positive control strain [DY3217 (+) in green] with strong termination due to the insertion of the *IMD2* intergenic terminator between the *GAL1* promoter and GFP is shown. A negative control strain [DY3218 (-) in red] with no terminator between the *GAL1* promoter and GFP is also shown. Results for experimental strains expressing Nab3Hrp1 (DY3197), Nab3Sup35 (DY3187), Nab3Ent2 (DY3246) or Nab3CT25 (DY4014) expressed from a plasmid as the sole Nab3 protein, are shown. (B) GFP fluorescence for DY3187 was monitored for colonies grown on SC leu^-^ solid medium supplemented with galactose and illuminated with the Dark Reader optical system (Clare Chemical Research, Inc.). (C) Flow cytometry of a Nab3-Sup35-expressing strain before (DY3187, blue) and after (DY3204, orange) conversion to the reporter-positive state (see text). A control strain was analyzed that lacks terminator function [DY3218 (-)]. (D) GFP fluorescence for DY3204 was monitored for colonies grown on SC leu^-^ containing galactose after illumination with the Dark Reader optical system (Clare Chemical Research, Inc.).

To test the ability of Nab3Hrp1, Nab3Sup35, and Nab3Ent2 to support transcription termination, we introduced a termination-reporter plasmid into the strains in which each chimera was the only source of Nab3. This reporter contains a strong terminator from the *IMD2* locus inserted between the *GAL1* promoter and the green fluorescent protein (GFP) reading frame. By using flow cytometry to score GFP abundance in living cells, this plasmid forms the basis of a sensitive assay for the termination of transcription by RNA polymerase II [6]. Strongly fluorescent cells are indicative of terminator readthrough when Nab3 activity is defective, whereas when termination is operative, GFP fluorescence is not detected (Fig 3A, “-”and “+”, respectively). In this assay, all three chimeras displayed nearly full terminator function. These results indicate that some heterologous LCDs restored termination factor activity to Nab3 lacking its C-terminal domain.

### The Nab3Sup35 chimera shows an epigenetic inheritance pattern

The strain with Nab3Sup35 showed a curious ability to generate infrequent variants that lost terminator function and started expressing GFP from the reporter plasmid, as seen by direct illumination of colonies on solid medium (Fig 3B). Over time, a plate of termination-competent, GFP-negative colonies produced sporadic, termination-defective GFP-positive colonies. Re-streaking cells from GFP-negative colonies led to the emergence of GFP-positive colonies at a rate similar to that of the original colony (~3x10^-3^). However, re-streaking of cells from colonies that were GFP-positive led to progeny colonies all of which were GFP-positive (Nab3Sup35*), demonstrating the heritability of this feature as would be expected for prion formation (Fig 3C and 3D). Since native Sup35 is known to form cytoplasmic aggregates in a prion domain-dependent manner, and this is concomitant with loss of Sup35’s function, we hypothesized that the Nab3Sup35 chimera could become converted into an inactive form, presumably as a cytoplasmic amyloid. In so doing, transcription termination on the reporter plasmid would be compromised, and GFP would be expressed. The strain with the Nab3Hrp1 chimera did not generate this type of spontaneous fluorescent colony, consistent with the idea that what was seen for Nab3Sup35 was a manifestation of Sup35’s well-characterized prion-forming behavior. Taken together, these data show that some, but not all, heterologous prion-like domains can substitute for the endogenous region of Nab3. Furthermore, the Nab3-fused Sup35 prion domain appears to retain its capacity to aggregate in living cells and display heritability while attached to Nab3. Interestingly, the rescuing pieces of protein share little or no primary sequence homology with Nab3’s LCD (S1 Fig), aside from low complexity, suggesting that rescue is achieved by another feature such as polymer formation, and not simply recognition of a specific primary sequence in the domain.

### An unbiased selection for sequences that substitute for Nab3’s LCD

Given the apparent lack of sequence specificity of the polypeptides that could rescue Nab3 function, we asked whether random DNA could provide essential sequences to Nab3Δ191 using yeast survival as the basis for an unbiased selection. A library of plasmids containing fragments of calf thymus DNA were fused to the stem of Nab3 used to generate the aforementioned chimera, and the plasmids were introduced into yeast with wildtype *NAB3* on a plasmid. Strains that could survive the loss of wildtype *NAB3* by virtue of living off of the Nab3 fusion protein that had acquired calf thymus sequences, were selected on FOA-containing medium. A plasmid that supported growth was recovered from this strain and sequenced showing that the rescuing piece of DNA was an 817bp insert derived from a non-coding portion of bovine chromosome 19 that adds 28 amino acids to the Nab3 stem before a stop codon is encountered (Fig 2 and S1 Fig). The resulting fused peptide (called “CT25”) is not glutamine- or asparagine-rich, a feature of many prion-like low complexity domains, although it does have a somewhat biased content of leucine, valine, and cysteine (14% each). This finding suggests there is a remarkable tolerance in the primary sequence needed to fulfill at least one essential function of the Nab3 RNA-binding protein, and its role can be served by many alternative sequences. However, not every fusion partner sufficed, indicating that simply ‘capping’ the end with any sequence is not what provides the essential function.

### Substitution of LCDs into Hrp1

**Fig 4:**
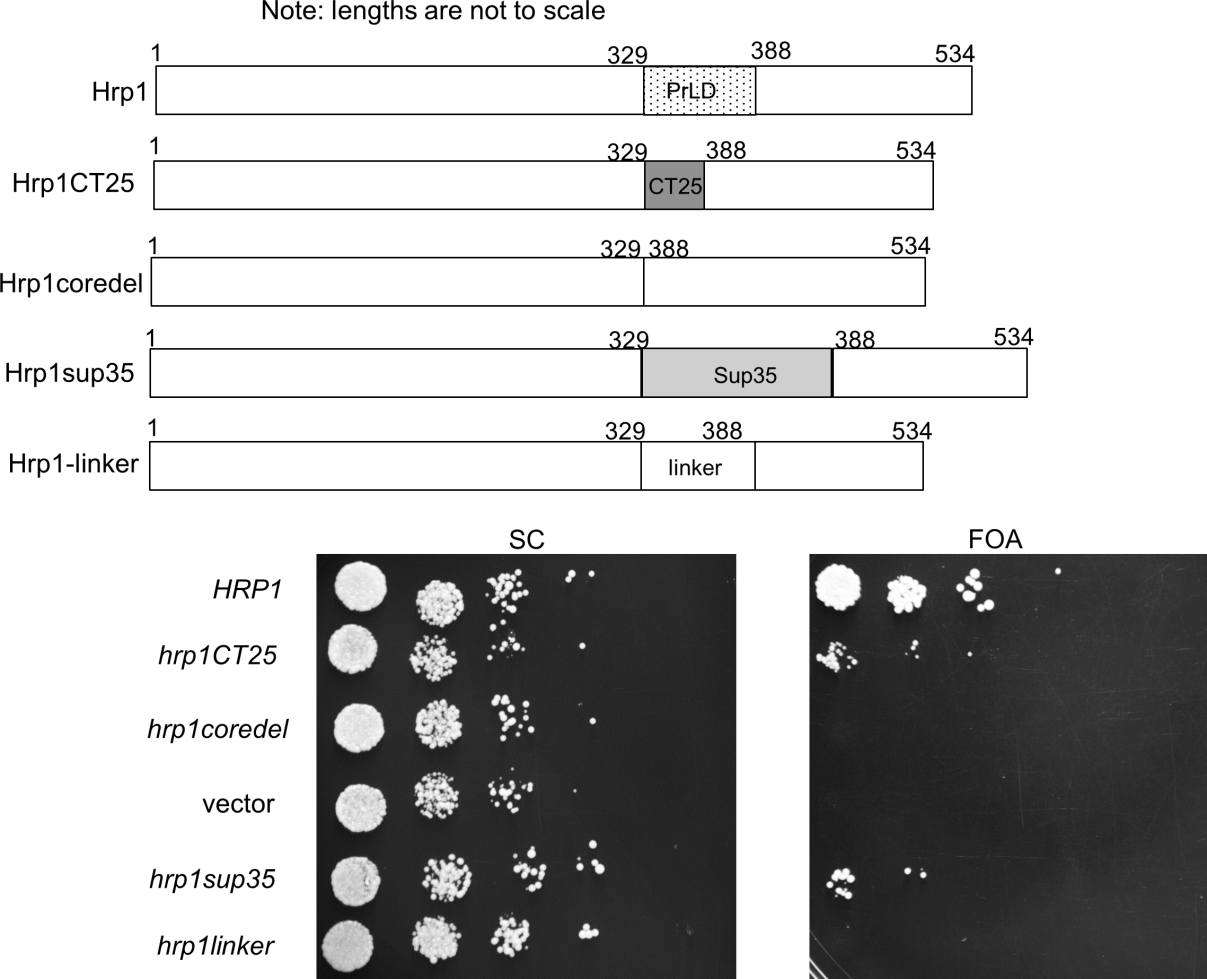
Viability of cells with Hrp1-chimeric proteins. A schematic depiction of the wildtype Hrp1 and the indicated LCD derivatives is shown at the top. Strains (DY388, DY387, DY389, DY1638, DY3242, and DY4500, from top to bottom) carrying the indicated plasmid-encoded proteins, as well as a *URA3*-marked plasmid with *HRP1*, were grown in SC leu^-^, diluted to OD_600_ of 0.05 and serially diluted ten-fold four times. Ten microliters of each dilution were spotted onto a single plate of the indicated solid media and cells were grown at 30°C.

**Fig 5:**
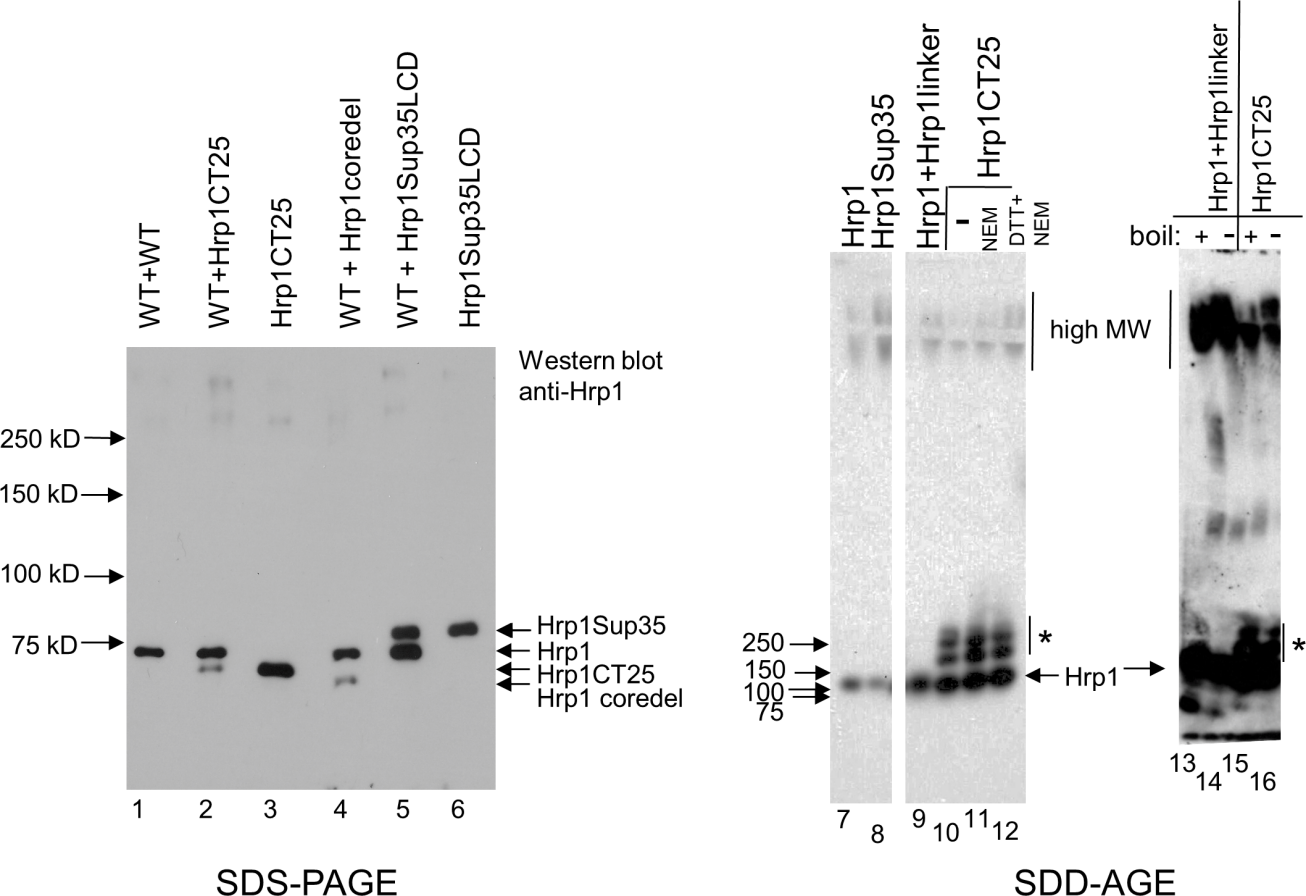
Western blotting and SDD-AGE analysis of Hrp1 and related chimera. Western blots were used to examine expression of Hrp1. Whole cell lysates from strains DY388, DY387, DY387F, DY389, DY3242, and DY3243 were boiled in loading buffer, resolved by SDS-PAGE, transferred to nitrocellulose, and probed with anti-Hrp1 antibody, as described in Methods. Pre-stained marker proteins were run in an adjacent lane and their sizes are indicated at left. To the right are SDD-AGE assays analyzing lysates from strains bearing the indicated Hrp1-derivatives (lane 7 = DY308, lane 8 = DY3243, lane 9 = DY4500, lanes 10–12 = DY387F, lanes 13 and 14 = DY388, lane 15 and 16 = DY387F), and developed with anti-Hrp1 antibody, as described in Methods. Lanes 7–12 (all lanes are derived from the same gel and filter) show that the oligomeric forms of Hrp1CT25 (“*”) are specific for that Hrp1 derivative, and that the proteins are relatively resistant to reducing agent. Lanes 13–16 show that the oligomers (“*”) and high molecular weight (MW) forms of Hrp1 and Hrp1CT25 are relatively resistant to boiling in SDS for 5 min before electrophoresis.

From a gene ontology perspective, Hrp1 appears similar to Nab3. Both are RNA-binding hnRNPs with LCDs that score highly as prion-like. Their LCDs form filaments *in vitro* [7, 23]. Both exist in a complex of proteins that interact with RNA polymerase II and both are involved in the termination of transcription by the enzyme [1, 5, 34]. First, we tested if Hrp1’s natural prion-like core domain (60 amino acids) was essential for cell viability as seen for the piece of Nab3 removed above. While wildtype *HRP1* on a plasmid could cover a deletion of a chromosomal copy of the gene, a plasmid encoding Hrp1 lacking its core LCD was not viable (Fig 4, “hrp1coredel”) even though the deletion-derivative could be expressed in yeast with wildtype *HRP1* (Fig 5, lane 4). Since the Hrp1 LCD, and the 28-amino acid sequence derived from bovine DNA could be swapped for Nab3’s essential domain, we tested if the bovine peptide could substitute for Hrp1’s core prion-like domain. Surprisingly, this sequence could also substitute for Hrp1’s core prion-like domain, where it was well expressed and enabled cell viability, although cells grew more slowly compared to those with wildtype *HRP1* (Fig 4, “*hrp1CT25*”, Fig 5, lane 3). Since 113-amino acids of Sup35’s low complexity prion-domain could be added to the Nab3 stem where it provided an essential function (Figs 1 and 2), we asked if the Sup35 prion-domain could do the same if exchanged for Hrp1’s core prion-like domain. Indeed, this construct also was expressed and rescued viability (Fig 4, “*hrp1sup35*”; Fig 5, lanes 5 and 6), demonstrating the remarkable cross-functionality of prion and prion-like domains, as well as the random sequence selected from mammalian DNA, when added to two different RNA-binding proteins in place of their native LCDs. (Sup35’s prion-domain is not essential for cell viability, so the viability test of exchanging native for heterologous sequences could not be applied to it.) As a negative control, we inserted 60 amino acids of the His_6_^-^ and S-Tag-containing linker from pET32a in place of the same length of Hrp1’s prion-like domain. This ‘*hrp1linker*’ derivative, while expressed in cells with wildtype *HRP1*, could not support viability as the sole form of Hrp1 (Fig 4). This controls for the possibility that the native Hrp1 sequence serves a ‘spacing’ function which might mean that replacing it with virtually any sequence would work. Hence, the LCD sequences we added are providing a specific property to the rest of the Hrp1 scaffold in order to cover function. It should be noted that the Hrp1 grafting experiments, in which the heterologous exchange was internal, differed from those for Nab3 where exchanges were made to its C-terminus.

The Hrp1 core prion-like domain can form aggregates when fused to a fluorescent protein [23]. To test if the full length Hrp1 protein, and our chimeric derivatives, could do so, we performed semi-denaturing detergent agarose electrophoresis (SDD-AGE) on cell lysates. This is a well-established assay for high molecular weight amyloid aggregates that are resistant to anionic detergent disruption [35]. Hrp1, Hrp1Sup35, and Hrp1CT25 all formed high molecular weight forms that barely migrated out of the starting wells (Fig 5, lanes 7–16). These aggregates were largely resistant to boiling (Fig 5, lanes 13 and 15 *vs* 14 and 16, respectively). Perhaps more interestingly, the Hrp1CT25 chimera displayed a new and unique set of forms which appeared to be dimer, trimer, and tetramers (Fig 5, “*”, lanes 10–12, 15, and 16). Although this rescuing mammalian sequence brings four cysteines into the protein that are not present in native Hrp1 (S2 Fig), reduction (1 mM dithiothreitol), or reduction and alkylation (1 mM dithiothreitol followed by 30 mM N-ethylmaleimide), did not dissociate the oligomeric forms of Hrp1CT25 (Fig 5, “*” lanes 10–12). These findings suggest that the selection of this particular heterologous sequence was due to its ability to form multimers, and potentially high molecular weight aggregates, and that the essential function of the LCDs from Hrp1 and presumably Nab3, is likely to be to promote oligomerization.

### Nab3’s essential C-terminus can be subdivided

**Fig 6:**
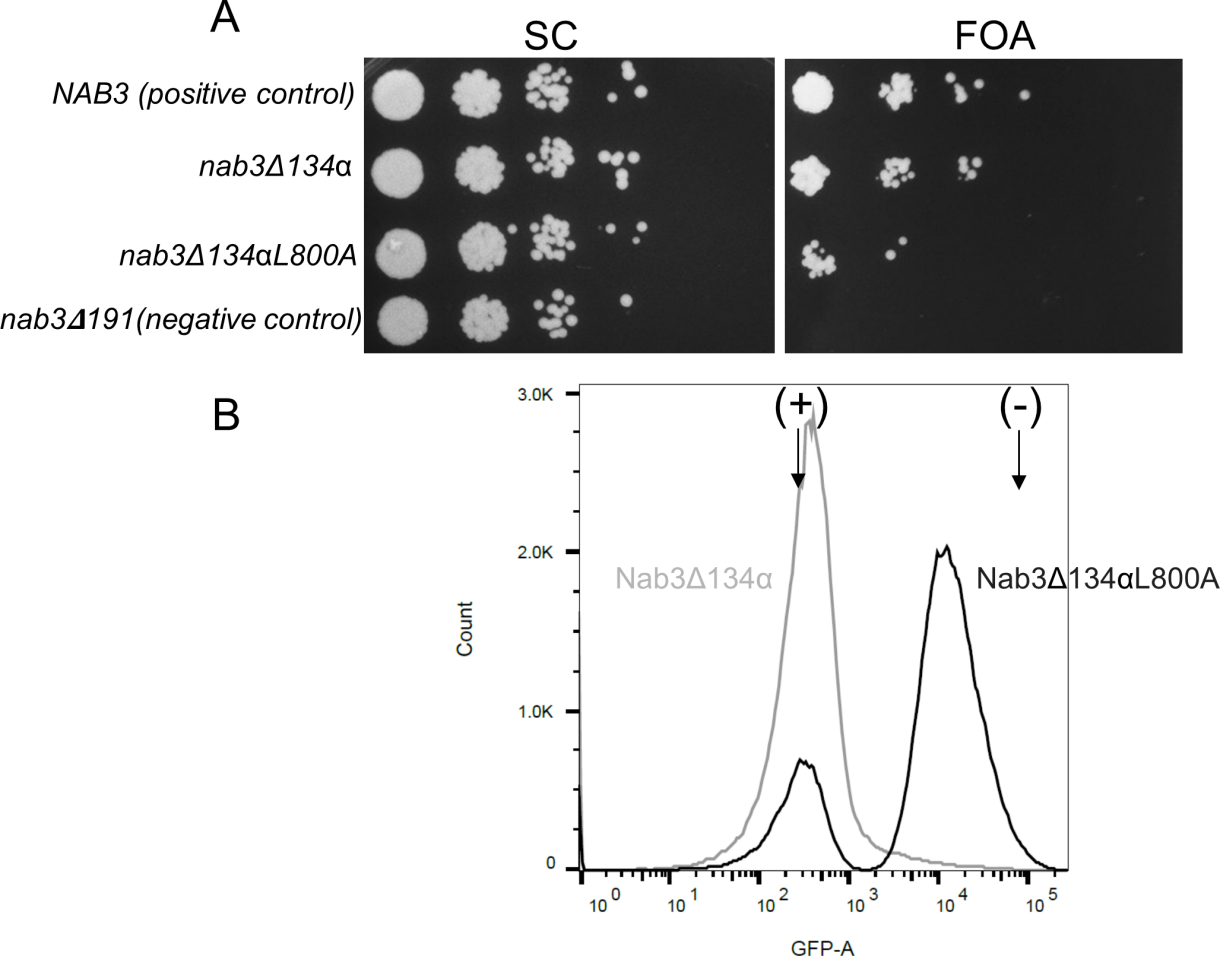
Bipartite nature of the Nab3 C-terminal essential region. A) Yeast strains containing a plasmid bearing wildtype *NAB3* on a URA3-marked plasmid and either the *nab3*Δ*134*α allele (strain DY353) or *nab3*Δ*134*α*L800A* allele (strain DY377) on *LEU2*-marked plasmids, were grown on complete medium (SC) or medium with FOA to test for viability after the loss of the former plasmid. Cells were diluted, spotted, and grown on a single plate of each indicated solid media at 30°C, along with a positive control (DY351; with wildtype *NAB3* on a *LEU2*-marked plasmid) and negative control strain with a non-viable allele (*nab3*Δ*191*) of *NAB3* (DY3183). B) The resulting shuffled FOA-resistant strains (DY359 [gray line] and DY379 [black line]) were tested for their termination competence as described in Materials and Methods. In lieu of reproducing the control strain peaks seen in Fig 3, only the position of the fluorescence maxima (arrows) for the termination-competent (+) (DY3217) and termination-defective (-) (DY3218) control strains are shown for reference.

The smallest portion of Nab3’s C-terminal region needed for viability was previously delimited to 134 amino acids as shown by plasmid shuffling or substitution of the deleted allele into the chromosome of a diploid followed by tetrad dissection ([22] and S2 Fig). The sequence contains two discernible components, the Q/P-rich region and the C-terminal-most 18 amino acids which has features of a leucine zipper and structural homology to a self-assembling segment of human hnRNP-C [36]. Deletion of this small peptide from Nab3’s terminus, or a subtle leucine to alanine substitution within it, are not lethal, but do slow growth and compromise Nab3’s termination function [6, 36]. To determine the contribution that these two elements provide to the region’s essential function, we added back the very C-terminal human hnRNP-C-homology sequence to Nab3 deleted for its terminal 134 residues. Surprisingly, this derivative was viable (Fig 6A “FOA”, nab3Δ134α), indicating that neither loss of a substantial part of the Q/P-rich domain, nor the C-terminal α-helix, are required for cell viability, however, both cannot be lost in order for the protein to remain functional. Hence, the Nab3 C-terminus has a bipartite nature based upon structural predictions and functional assays. We asked if the C-terminal human hnRNP-C-homology domain could support the loss of the Q/P domain when the former contained the previously defined L800A mutation known to compromise termination. The mutation compromised but did not abolish the homology region’s ability to protect against loss of the Q/P sequences (Fig 6B, “nab3Δ134αL800A”). We confirmed that the L800A mutation in this context compromised termination using the flow cytometry reporter assay (Fig 6B). These results suggest that the rescuing effect is related to the role of the domain in termination and that the LCD-chimeras described above appear to provide this dual property of the native sequence when fused to the Nab3 stem.

## Discussion

Numerous cellular RNA-binding proteins contain LCDs that can self-assemble. This enables them to form dynamic compartments in cells in which RNA metabolism is sequestered. In this report, we set out to determine the interoperability of LCDs in the yeast proteome to gain insight into their role in the context of a well-studied RNA-binding protein. We exploited the fact that Nab3 contains an essential C-terminal region, which permitted us to test heterologous LCDs whose sequence and potential for self-assembly derive from their relatedness to yeast prions. The ability to rescue viability by the chimeras was followed by functional tests of termination. A starting set of six LCDs were derived from proteins of varying functions, four RNA-binding proteins, two canonical yeast prions, and one membrane associated epsin-like protein. Three of these resulted in fusion proteins that could supply function to live yeast. The chimeric proteins showed near normal termination activity in our assays. Interestingly, the viable fusions came from proteins with disparate biological roles. Hrp1 is a component of cleavage factor 1 (CF1), and is a nuclear RNA-binding protein necessary for the hydrolysis and polyadenylation of pre-mRNA’s 3’ ends. It is also involved in termination of short non-coding RNAs and autoregulation of its own mRNA. Thus, it is similar to Nab3’s biochemical and biological functions. In contrast, Sup35 is a cytoplasmic translation factor that associates with ribosomes and is a canonical yeast prion and Ent2 is a cytoplasmic epsin-like protein involved in the endocytic pathway that has no obvious direct interactions with RNA or RNA-binding proteins. The Sup35 prion domain is not essential for its role in translation, but in a sense, it becomes ‘essential’ when added to Nab3 in the aforementioned grafting experiment.

A simple model suggests that any amyloid-forming LCD can substitute for any other such domain, if the only critical function it provides is that of self-assembly. That clearly is not the case here. Alternatively, the domains might also encode specific protein-protein docking sites for which their amino acid primary structure would be important. That does not seem to be the case here either, based on the surprising finding that distinct LCDs could cover for one another, albeit not universally. Proteins with similar biological functions might be expected to have cross-compatible LCD’s. Yet neither termination-related protein Rat1 nor Pcf11 had amyloid-forming LCDs that could supply function to the Nab3 stem.

What properties create the specificity of function within the family of LCDs? Table 1 shows the composition of the domains studied here with respect to those amino acids over-represented in amyloid-forming prion-like domains. The seven amino acids glutamine, asparagine, proline, serine, tyrosine, glycine, and methionine compose 65–90% of each of the yeast domains we examined (Table 1). In general, successful LCDs were no closer to the amino acid composition of the piece of the LCD removed from Nab3, than were those LCDs that failed the test (Table 1). For example, Pcf11’s LCD (39% Q) could not be swapped for the region removed from Nab3 (27%Q) in our experiments, whereas the 15% Q domain from Hrp1 could. There has been a lot of attention paid to Q-rich domains because of their ability to assemble into amyloid filaments and their involvement in human disease. The abundance and length of Q-tracts in proteins is important for their aggregation properties. This is true for Nab3 where the glutamines in Nab3’s LCD are important since a reduction in Q-content by mutation destroys the LCD’s ability to form an amyloid polymer and leads to cellular lethality [8]. A similar discordance was seen for proline content where Nab3’s 18%-proline domain could be replaced by Hrp1’s which was only 4% proline. Likewise, the ability of an LCD to rescue is not correlated with the LCD’s: 1) asparagine content, 2) proline plus glutamine content, or 3) glutamine plus asparagine content, as can be seen in Table 1. Our data are consistent with a model in which a diffuse and redundant amino acid composition that leads to the host protein’s assembly, is the important property conferred to the Nab3 stem. However, different LCDs must be able to assemble into distinct conformers, assemble to different extents, or otherwise generate different structures to which other proteins might join. This model is attractive because it obviates the need to postulate that a specific primary sequence is needed, while proposing that not all assembly/polymerization functions of LCDs are equivalent. However, without additional experimentation, we cannot resolve the specific and complex primary and secondary structural characteristics that are responsible for LCD function in the context of Nab3. In any case, there appear to be subclasses of LCDs, the effectiveness of which cannot be predicted by the function of their host protein. One possibility is that each category of LCD may be a determinant of its host proteins’ ability to localize to a specific subcellular location, *e*.*g*. during recruitment of RNA-binding proteins to stress granules.

**Table 1:** Frequency in proteins studied here of the residues commonly over-represented in amyloid-forming LCDs.

	Length	%Q	%N	%S	%P	%Y	%G	%M	%PQ	%QN	%QNP	%QNSPYGM
Nab3^609-803^	194	27	6	10	18	5	7	4	45	6	51	77
**Hrp1**^**329-388**^	**60**	**15**	**22**	**0**	**5**	**10**	**17**	**15**	**20**	**22**	**42**	**83**
**Sup35**^**2-114**^	**113**	**28**	**18**	**4**	**4**	**18**	**17**	**0**	**33**	**18**	**50**	**88**
**Ent2**^**540-599**^	**60**	**65**	**5**	**0**	**10**	**8**	**0**	**2**	**75**	**5**	**80**	**90**
Pcf11^208-287^	80	39	8	8	1	4	3	4	40	8	48	65
Rat1^933-1006^	74	7	30	15	3	12	10	0	10	37	40	77
Rnq1^124-405^	282	25	15	17	1	4	17	2	26	15	41	81
CT25	28	4	0	4	0	0	7	4	4	0	4	18

The regions of the indicated yeast proteins (and the bovine sequence CT25 described herein) were analyzed for their content of the amino acids commonly enriched in amyloid-forming, low complexity domains. The fraction of residues that are glutamine (Q), asparagine (N), serine (S), proline (P), tyrosine (Y), glycine (G), methionine (M), proline+glutamine (PQ), glutamine+asparagine (QN), glutamine+asparagine+proline (QNP), or glutamine+asparagine+serine+proline+tyrosine+glycine+methionine (QNSPYGM) was calculated and rounded to the nearest percent. The bold sections are those yeast LCDs that could substitute for Nab3’s, those in plain text did not. The bovine sequence is shown at the bottom.

The viable, chimeric Nab3 proteins were capable of operating the *IMD2* terminator that regulates Nab3. Flow cytometric analysis found near wild type levels of GFP expression, implying that the heterologous LCDs efficiently complement Nab3’s function in our termination assay. While the exact *in vivo* function of Nab3’s LCD is unclear, the findings here support prior suggestions [7, 36] that it mediates either homotypic or heterotypic protein-protein interactions. It is interesting that the LCDs of proteins that localize to different compartments of the cell and possess disparate functions, can mediate these same interactions.

The spontaneous emergence of cross generationally stable, termination defective yeast containing the Nab3Sup35 chimera was an unexpected outcome. The finding suggests that Nab3Sup35 is undergoing some prion-like event resulting in stably heritable, termination defective yeast. The phenomenon is analogous to the molecular genetic protein nullification tool called ‘anchor-away’ which is a method of re-localizing to the cytoplasm, and thereby inactivating, a nuclear protein by genetically fusing it to a protein that binds a cytoplasmic counterpart [37]. It is also reminiscent of the aberrant aggregation-dependent cytosolic migration seen for nuclear RNA-binding proteins with LCDs such as TDP-43, fused in sarcoma (FUS), or hnRNP-A1 which aberrantly accumulate in the cytosol in diseases such as amyotrophic lateral sclerosis [17].

Since the heterologous LCD’s shared little primary sequence similarity, we performed an unbiased screen using calf thymus DNA as a source of coding sequence to search for a fusion protein capable of sustaining viability. This initial proof-of-principle investigation used bovine genomic DNA as a rich source of complex random sequence. A more comprehensive analysis could yield the frequency with which such ‘winners’ might be found. Nevertheless, the clone we pursued was intriguing, even though it was not predicted to be disordered as expected from the prion-like sequences we employed from yeast. The evidence that it was functional was supported by its ability to rescue the lethality of the loss of either Nab3 or Hrp1’s LCD and its ability to form novel higher order assemblies. The encoded peptide did not score well as a prion, due in part to the relative paucity of Q and N residues (4% and 0%, respectively) and relatively high content of charged residues (29%). Although, it does show somewhat of a biased sequence makeup, significant hydrophobicity, and low complexity with a repeated set of cysteines, valines, and leucines composing 43% of the sequence. It seems plausible, if not likely, that the sequence rescues Nab3 function by facilitating Nab3 polymerization, although it may do so in a manner distinct from that of prion-like LCDs. In any case, the fact that assembly intermediates are visible with the Hrp1CT25 chimera helps validate that the screen for cell viability by generating Nab3-fusion proteins produced domains whose main role is to provide assembly interactions and maybe little else. This in turn supports the model that one function of at least some LCDs in RNA-binding proteins is to promote polymerization.

We have previously characterized a portion of the C-terminus of Nab3 adjacent to the LCD that appears to be an α-helix based upon its structural homology to human hnRNP-C [36]. Loss of this helix, or even subtle alterations to its sequence such as the L800A substitution, had profound impacts on growth and termination, although they are not lethal. Similarly, removal of a large portion of the Q/P-rich region alters growth and termination but that alone is not lethal (Fig 6, “nab3Δ134α”). While either the Q/P-rich domain or the human hnRNP-C structural homology segment are sufficient for viability, loss of both is lethal, implying functional redundancy between the two domains. The fact that the C-terminal α-helical domain’s ability to ameliorate the loss of a large stretch of Nab3’s LCD is reduced when it contains the L800A mutation that affects termination, suggests that the contribution of this region to viability is related to its role in transcription termination.

This report expands our understanding of a well characterized termination factor, Nab3, and the role of its LCD in termination function. It also suggests that while a simple primary sequence does not determine an LCD’s function, there does appear to be specialization of function within the family of LCDs. This confirms the important role of these domains in biology and shows that the LCDs are more complex than previously appreciated.

## Supporting information

S1 Fig Sequences of proteins studied here. The entire Nab3 sequence is shown with position 191 identified. Nab3^1-191^ was used as the ‘stem’ for the addition of LCDs from the remaining group of proteins. The Nab3 italicized residues are the prion-like domain based on Alberti *et al*.’s algorithm [23]. The bold sequence is the region of Nab3 with structural homology to human hnRNP-C’s α-helix [36]. LCDs from yeast are listed with their native coordinates. The scrambled Hrp1 sequence and sequences from the bovine genome described here are also listed. The glutamine, asparagine, and proline residues are color coded red, green, and blue, respectively.
(TIF)

S2 Fig Tetrad dissection of a diploid with wildtype *NAB3* and *nab3Δ134* alleles. The arrows indicate six columns each from different tetrads. The rows are the four positions in which spores were deposited.
(TIF)

S1 Table Oligonucleotides used. (PDF)

S2 Table Yeast strains studied here. (PDF)
