# Supplementary figures and images for: The hnRNP-like Nab3 termination factor can employ heterologous prion-like domains in place of its own essential low complexity domain
Source: PLoS One. 2017 Oct 12;12(10):e0186187. doi: 10.1371/journal.pone.0186187 (PMC5638401; doi:10.1371/journal.pone.0186187)

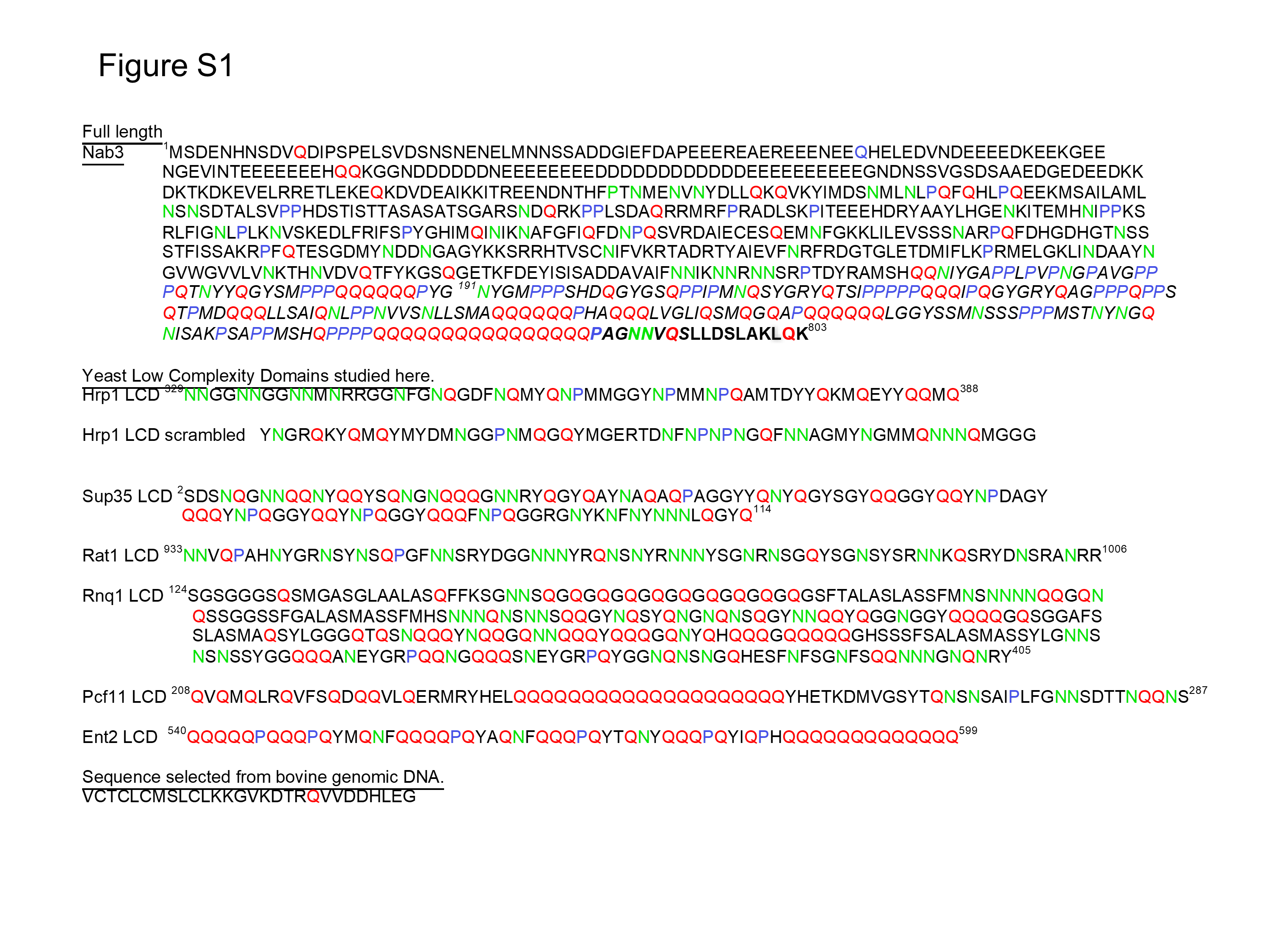

Supplement: S1 Fig — The entire Nab3 sequence is shown with position 191 identified. Nab31-191 was used as the ‘stem’ for the addition of LCDs from the remaining group of proteins. The Nab3 italicized residues are the prion-like domain based on Alberti et al.’s algorithm [23]. The bold sequence is the region of Nab3 with structural homology to human hnRNP-C’s α-helix [36]. LCDs from yeast are listed with their native coordinates. The scrambled Hrp1 sequence and sequences from the bovine genome described here are also listed. The glutamine, asparagine, and proline residues are color coded red, green, and blue, respectively. (TIF) [file pone.0186187.s001.tif]

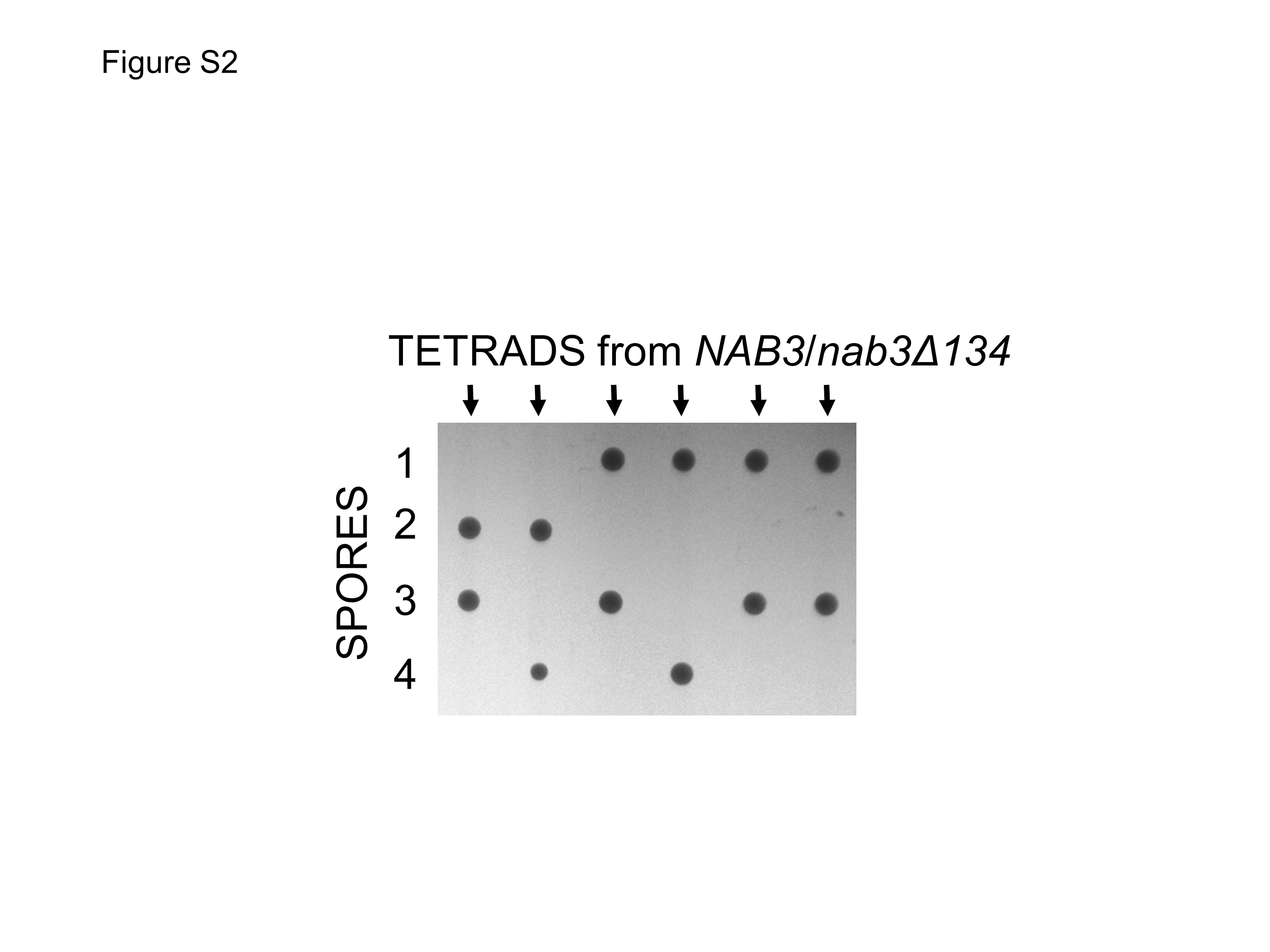

Supplement: S2 Fig — The arrows indicate six columns each from different tetrads. The rows are the four positions in which spores were deposited. (TIF) [file pone.0186187.s002.tif]
