# Supplementary material for: The hnRNP-like Nab3 termination factor can employ heterologous prion-like domains in place of its own essential low complexity domain
Source: PLoS One. 2017 Oct 12;12(10):e0186187. doi: 10.1371/journal.pone.0186187 (PMC5638401; doi:10.1371/journal.pone.0186187)
Supplement: S1 Table — (PDF) [file pone.0186187.s003.pdf]

RNQ1 F	5’ ATATATCATATGGTAGTGGTTCTGGCGGC 3’
RNQ 1 R	5’ ATATATCTCGAGTCAGTAGCGGTTCTGGTTGC 3’
PCF11 F	5’ ATATATCATATGGTCAAGTTCAAATGCAACTA AGGC 3’
PCF11 R	5’ ATATATCTCGAGCTAAGAATTCTGTTGGTTCGTTGT ATC 3’
RAT1 F	5’ ATATATCATATGGTAATAATGTCCAACCCGCC 3’
RAT1 R	5’ ATATATCTCGAGCTAACGCCTATTTGCTCTTGAA 3’
HRP1 F	5’ ATATATCATATGGTAACAATGGTGGTAACAATGG 3’
HRP1 R	5’ ATATATCTCGAGCTATTGCATTTGTTGGTAATATTCTTG 3’
SUP35 F	5’ ATATCATATGGTTCGGATTCAAACCAAGGC 3’
SUP 35 R	5’ ATATCTCGAGCTATTGATATCCTTGCAAATTGTTATTG 3’
