# Supplementary material for: The hnRNP-like Nab3 termination factor can employ heterologous prion-like domains in place of its own essential low complexity domain
Source: PLoS One. 2017 Oct 12;12(10):e0186187. doi: 10.1371/journal.pone.0186187 (PMC5638401; doi:10.1371/journal.pone.0186187)
Supplement: S2 Table — (PDF) [file pone.0186187.s004.pdf]

| | | |
|---|---|---|
| DY307 | *MATa hrp1::HIS3 ura3 ade2 ade8 his3 leu2 lys1* Trp$^{-}$ [pRS315-*HRP1* (*LEU2*)] [pRS316-HRP1 *(URA3)*] | this study |
| DY308 | *MATa hrp1::HIS3 ura3 ade2 ade8 his3 leu2 lys1* Trp$^{-}$ [pRS316-*HRP1 (URA3)*] | this study |
| DY318 | *MATa hrp1::HIS3 ura3 ade2 ade8 his3 leu2 lys1* Trp$^{-}$ [pRS316-*HRP1 (URA3)*] [pRS315-HRP1coredel (*LEU2*)] | this study |
| DY351 | *MATα ura3Δ0 his3Δ1 leu2Δ0 nab3Δ0::kanM*X [pRS316-*NAB3* (*URA3*)] [pRS315-*NAB3* (*LEU2*)] | this study |
| DY353 | *MATα ura3Δ0 his3Δ1 leu2Δ0 nab3Δ0::kanMX* [pRS316-*NAB3* (*URA3*)] [pRS315-Nab3*Δ*134α (*LEU2*)] | this study |
| DY359 | *MATα ura3Δ0 his3Δ1 leu2Δ0 nab3Δ0::kanM*X [pRS315-Nab3*Δ*134α (*LEU2*)] | this study |
| DY377 | *MATα ura3Δ0 his3Δ1 leu2Δ0 nab3Δ0::kanMX* [pRS316-NAB3 (*URA3*)] [pRS315-Nab3Δ134α-L800A(*LEU2*)] | this study |
| DY379 | *MATα ura3Δ0 his3Δ1 leu2Δ0 nab3Δ0::kanMX* [pRS315-Nab3Δ134α-L800A(*LEU2*)] | this study |
| DY387 | *MATa hrp1::HIS3 ura3 ade2 ade8 his3 leu2 lys1* Trp$^{-}$ [pRS316-*HRP1 (URA3)*] [*pRS315Hrp1coredelCT25 (LEU2)*] | this study |
| DY387F | *MATa hrp1::HIS3 ura3 ade2 ade8 his3 leu2 lys1* Trp$^{-}$ [pRS315-Hrp1coredelCT25 *(LEU2)*] | this study |
| DY388 | *MATa hrp1::HIS3 ura3 ade2 ade8 his3 leu2 lys1* Trp$^{-}$ [pRS316-*HRP1 (URA3)*] *[pRS315-HRP1(LEU2)*] | this study |
| DY389 | *MATa hrp1::HIS3 ura3 ade2 ade8 his3 leu2 lys1* Trp$^{-}$ [pRS316-*HRP1 (URA3)*] *[pRS315-Hrp1coredel (LEU2)*] | this study |
| DY1638 | *MATa hrp1::HIS3 ura3 ade2 ade8 his3 leu2 lys1* Trp$^{-}$ [pRS316-*HRP1 (URA3)*] [pRS315 *(LEU2)*] | this study |
| DY3033 | *MATα ura3Δ0 his3Δ1 leu2Δ0 nab3Δ0::kanMX* [pRS315-Nab3FL (*LEU2*)] | Loya et al. 2012 |
| DY3034 | *MATα ura3Δ0 his3Δ1 leu2Δ0 nab3Δ0::kanMX* [pRS315-Nab3 (*LEU2*)] | Loya et al. 2012 |
| DY3111 | *MATα ura3Δ0 his3Δ1 leu2Δ0 nab3Δ0::kanMX* [pRS316-*NAB3* (*URA3*)] | O'Rourke & Reines, 2016 |
| DY3134 | *MATα ura3Δ0 his3Δ1 leu2Δ0 nab3Δ0::kanMX* [pRS316-*NAB3 (URA3)*] [pRS315-Nab3*Δ*134 (*LEU2*)] | this study |
| DY3182 | *MATα ura3Δ0 his3Δ1 leu2Δ0 nab3Δ0::kanMX* [pRS315-Nab3Sup35 (*LEU2*)] | this study |
| DY3183 | *MATα ura3Δ0 his3Δ1 leu2Δ0 nab3Δ0::kanMX* [pRS316-*NAB3* (*URA3*)] [pRS315-Nab3*Δ*191STOP (*LEU2*)] | this study |
| DY3184 | *MATα ura3Δ0 his3Δ1 leu2Δ0 nab3Δ0::kanMX* [pRS316-*NAB3 (URA3)*] [pRS315-Nab3Rat1 (*LEU2*)] | this study |
| DY3185 | *MATα ura3Δ0 his3Δ1 leu2Δ0 nab3Δ0::kanMX* [pRS316-*NAB3 (URA3)*] [pRS315-Nab3Pcf11 (*LEU2*)] | this study |
| DY3186 | *MATα ura3Δ0 his3Δ1 leu2Δ0 nab3Δ0::kanMX* [pRS316-*NAB3 (URA3)*] [pRS315Nab3Rnq1 (*LEU2*)] | this study |
| DY3187 | *MATα ura3Δ0 his3Δ1 leu2Δ0 nab3Δ0::kanMX* [pRS315-Nab3Sup35 (*LEU2*)] [pREFGFPKan (*URA3*)] | this study |

| | | |
|---|---|---|
| DY3193 | *MATα ura3Δ0 his3Δ1 leu2Δ0 nab3Δ0::kanMX* [pRS316-*NAB3 (URA3)*] [pRS315-Nab3Hrp1 (*LEU2*)] | this study |
| DY3196 | *MATα ura3Δ0 his3Δ1 leu2Δ0 nab3Δ0::kanMX* [pRS315-Nab3Hrp1 (*LEU2*)] | this study |
| DY3197 | *MATα ura3Δ0 his3Δ1 leu2Δ0 nab3Δ0::kanMX* [pRS315-Nab3Hrp1 (*LEU2*)] [pREFGFPKan *(URA3)*] | this study |
| DY3204 | *MATα ura3Δ0 his3Δ1 leu2Δ0 nab3Δ0::kanMX* [pRS315-Nab3Sup35]* (*LEU2*) [pREFGFPKan *(URA3)*] *epigenetic change to GFP expression (see text) | this study |
| DY3210 | *MATa/α ade5-1/ade5-1 his7-2/his7-2 leu2-3/leu2-3 trp1-289/trp1-289 ura3-52/ura3-52 NAB3/nab3Δ134* | this study |
| DY3213 | *MATα ura3Δ0 his3Δ1 leu2Δ0 nab3Δ0::kanMX* [pRS316-*NAB3 (URA3)*] [pRS315-Nab3Hrp1scr (*LEU2*)] | this study |
| DY3217 | *MATα ura3Δ0 his3Δ1 leu2Δ0 nab3Δ0::kanMX* [pRS315-Nab3 (*LEU2*)] [pREFGFPKan (*URA3*)] | this study |
| DY3218 | *MATα ura3Δ0 his3Δ1 leu2Δ0 nab3Δ0::kanMX* [pGalGFP-Kan (*URA3*)] [pRS315-*NAB3* (*LEU2*)] | this study |
| DY3242 | *MATa hrp1::HIS3 ura3 ade2 ade8 his3 leu2 lys1* Trp$^-$ [pRS316-HRP1 *(URA3)*] [pRS315 Hrp1Sup35LCD *(LEU2)*] | this study |
| DY3243 | *MATa hrp1::HIS3 ura3 ade2 ade8 his3 leu2 lys1* Trp$^-$ [pRS315 Hrp1Sup35LCD *(LEU2)*] | this study |
| DY3244 | *MATa hrp1::HIS3 ura3 ade2 ade8 his3 leu2 lys1* Trp$^-$ [pRS315 Nab3ENT2LCD *(LEU2)*] [pRS316-*NAB3 (URA3)*] | this study |
| DY3245 | *MATa hrp1::HIS3 ura3 ade2 ade8 his3 leu2 lys1* Trp$^-$ [pRS315 Nab3ENT2LCD *(LEU2)*] | this study |
| DY3246 | *MATa hrp1::HIS3 ura3 ade2 ade8 his3 leu2 lys1* Trp$^-$ [pRS315 Nab3ENT2LCD *(LEU2)*] [pREF-GFPKan(URA3)] | this study |
| DY3912 | *MATα ura3Δ0 his3Δ1 leu2Δ0 nab3Δ0::kanMX* [pRS315-Nab3Δ134α (*LEU2*)] [pGAL-REF-GFP *(URA3)*] | this study |
| DY3913 | *MATα ura3Δ0 his3Δ1 leu2Δ0 nab3Δ0::kanMX* [pRS315-Nab3Δ134αL800A (*LEU2*)][pGAL-REF-GFP *(URA3)*] | this study |
| DY4001 | *MATα ura3Δ0 his3Δ1 leu2Δ0 nab3Δ0::kanMX* [pRS316-*NAB3 (URA3)*] [pRS315-Nab3CT25 (*LEU2*)] | this study |
| DY4002 | *MATα ura3Δ0 his3Δ1 leu2Δ0 nab3Δ0::kanMX* [pRS316-*NAB3 (URA3)*] [pRS315-Nab3Sup35 (*LEU2*)] | this study |
| DY4004 | *MATα ura3Δ0 his3Δ1 leu2Δ0 nab3Δ0::kanMX* [pRS315-Nab3CT25 (*LEU2*)] | this study |
| DY4006 | *MATα ura3Δ0 his3Δ1 leu2Δ0 nab3Δ0::kanMX* [pRS315-Nab3hrp1scr (*LEU2*)] | this study |
| DY4014 | *MATα ura3Δ0 his3Δ1 leu2Δ0 nab3Δ0::kanMX* [pRS315-Nab3CT25 (*LEU2*)] [pREF-GFPKan(*URA3*)] | this study |
| DY4500 | *MATa hrp1::HIS3 ura3 ade2 ade8 his3 leu2 lys1* Trp$^-$ [pRS316-*HRP1 (URA3)*] [pRS315-Hrp1-linker (*LEU2*)] | this study |
| PSY818 | *MATa hrp1::HIS3 ura3 ade2-1 ade8 his3 leu2-3 lys1* Trp$^-$ [pRS315-HRP1 (*LEU2*)] | Henry *et al*. 2003 |
| YH990 | *MATa/α ade5-1/ade5-1 his7-2/his7-2 leu2-3/leu2-3 trp1-289/trp1-289 ura3-52/ura3-52* | Morrison *et al*. 1991 |
